# A cost effective analysis of fixed-dose combination of dutasteride and tamsulosin compared with dutasteride monotherapy for benign prostatic hyperplasia in Nigeria: a middle income perspective; using an interactive Markov model

**DOI:** 10.1186/s12885-016-2431-x

**Published:** 2016-07-07

**Authors:** Emeka I. Udeh, Chimaobi G. Ofoha, David A. Adewole, Ikenna I. Nnabugwu

**Affiliations:** Department of Surgery, Faculty of medicine, College of Medicine, University of Nigeria Enugu campus, Enugu, Nigeria; Department of Surgery, University of Nigeria Teaching Hospital Ituku-Ozalla Enugu, Enugu, Nigeria; Department of Community Medicine, Bowen University/Bowen University Teaching Hospital, Iwo/Ogbomoso, Nigeria; Division of Urology, Department of Surgery, Jos University Teaching Hospital, Jos, Nigeria

**Keywords:** Cost effectiveness analysis, Dutasteride monotherapy, Fixed dose combination of dutasteride- tamsulosin, Nigerian men, Benign prostatic hyperplasia

## Abstract

**Background:**

The number of Nigerian men presenting with benign prostatic hyperplasia is on the rise because of increase awareness about the ailment. With the renewed effort by the national health insurance scheme to cover the informal sector, it becomes imperative to determine the cost implication for managing Benign Prostatic Hyperplasia (BPH) and the cost effective drug combination to be adopted. The objective of this study is to estimate cost effective analysis (CEA) of fixed -dose combination of dutasteride and tamsulosin compared with dutasteride monotherapy from the health service provider perspective design.

**Methods:**

An interactive Markov’s model was used to generate incremental cost per QALY and incremental cost per life years gained. 2.9 million Men who were 50 years of age were fed into the model. The outcome measures included: costs of drug treatment, consultation, acute urinary retention (AUR), transurethral resection of prostate (TURP), hospitalisation post TURP, and quality adjusted life years (QALYs), incremental cost per life years gained, and incremental cost per QALY gained.

**Results:**

Fixed-dose combination of dutasteride and tamsulosin (FDCT) produced an Incremental cost-effectiveness ratios of US$1481.92 per Quality adjusted for life-years saved.

**Conclusion:**

Universal FDCT provision for Nigeria has major economic implications. This study in the context of its limitations has demonstrated the cost effectiveness of FDCT for the long term treatment of patients with moderate to severe BPH from the perspective of a developing country. Currently, there are few studies available to give economic data evidence to policy makers in Nigeria which is applicable to developing countries with similar economies. As such, the findings in this study will be relevant to policy makers in these countries.

## Background

Social Health Insurance Scheme was introduced in Nigeria about a decade ago. It commenced with the enrolment of the formal sector workers, however, there is plan to extend it to the informal sector. However, the extent of coverage is still limited. Some non-communicable diseases are yet to be fully covered. Therefore, it became imperative to explore cost effective measures to ensure that certain ailments not yet covered are considered to be included in the benefit package.

While combination therapy has gained acceptance in certain treatment settings in various countries [1–3] questions regarding its cost effectiveness remains: Is there any additional benefit in introducing fixed dose combination therapy when compared with 5-alpha reductase inhibitors alone? Are the benefits of fixed dose combination therapy worth the additional expense of the second prescription medication?

In addition, Nigeria being a middle income country (gross domestic product of $574 billion) shares the same economic health challenges as most countries in sub-Saharan Africa and Asia that may have same economy or poorer economic status. The cost effective measure derived from this study will be relevant to policy makers in such economies. This will enhance a robust health insurance scheme with a comprehensive package for the informal sector.

About 22.3 % of the male population in Nigeria are diagnosed annually to have benign prostatic hyperplasia (BPH) [4]. Currently, about 3,000,000 men are 50 years old based on the projections from the 2006 population census [5]. This number is likely to increase as life expectancy improves with improving economic status of the population. The newly rebased gross domestic product (GDP) could be an evidence of an improving economy in the country.

BPH manifest through lower urinary tract symptoms (LUTS). If untreated, it can progress to complications such as obstructive nephropathy, acute urinary retention (AUR) and recurrent urinary retention [6, 7]. The main reason for treating BPH is to improve symptoms and reduce risk of progression. For patients with mild symptoms; watchful waiting is the treatment option. However, patients with moderate or severe BPH will require medical treatment [8].

BPH currently is being treated with a combination of tamsulosin and dutasteride (combination therapy) [9–11]. As the disease progresses, there may be need to offer either minimally invasive therapy (transurethral resection of the prostate) [12] or open prostatectomy. Studies have demonstrated the efficacy of these combination therapy (CT) in the management of BPH [13]. The current pharmacological treatment protocol for LUTS caused by BPH are alpha blockers (AB) such as tamsulosin and 5-alpha reductase inhibitors (5-ARI) such as dutasteride. Tamsulosin relaxes the smooth muscles of the prostate and bladder neck thereby increasing urine flow. Dutasteride reduces the vascularity and size of the prostate by inhibiting formation of intra-prostatic dihydrotestosterone [13, 14].

The Nigerian standard treatment guideline recommended alpha adrenergic blockers for the relief of symptoms in patients without prostate enlargement [15]. Though, this treatment option does not affect progression of disease. Also, ARI as either monotherapy or in combination with AB are recommended for patients with symptomatic BPH who have prostate enlargement. Combination therapy effectively reduce risk of disease progression.

On the other hand, the efficacy of dutasteride monotherapy in the management of BPH has been proven [10, 14, 16]. A 4-year randomised controlled trial designed to evaluate the effectiveness of tamsulosin or dutasteride monotherapy compared to combination of Avodart and Tamsulosin; showed that combination therapy significantly reduced the relative risk of AUR and surgery compared to tamsulosin by 67.6 and 70.6 % respectively and 18.3 % for AUR and 31.1 % for surgery compared to dutasteride [13].

Recently, there was a shift from combination therapy of tamsulosin and dutasteride to a fixed- dose combination of tamsulosin and dutasteride (FDCT). This shift, primarily was intended to reduce cost and also make drug use more convenient for the patients who will be on these drugs for a long time. With the fixed dose, the patient takes only a tablet daily compared to two tablets previously. The available brand in Nigeria is Duodart produced and marketed by GSK pharmaceutical company.

The objective of this study was to estimate the long term cost effectiveness of fixed- dose combination of dutasteride and tamsulosin compared with dutasteride monotherapy in the treatment of patients who present with moderate or severe BPH from the health service provider perspective of Nigeria; a middle income country.

## Methods

### The model structure

A discrete Markov model was developed to calculate the costs, health benefits and cost effectiveness of FDCT versus DM and to simulate the progression of BPH with a cycle length of 1 year. The Markov model was selected because BPH is a chronic condition with repeated clinical events.

The model was run for ten (1-year) cycles and consists of six mutually exclusive health states: (1) healthy; (2) mild BPH symptoms; (3) moderate BPH symptoms; (4) severe BPH symptoms; (5) Acute urinary retention; (6) TURP and (7) death. The symptom severity were determined by the international prostate symptom score (IPSS) and presence of complications such as acute urinary retention. Based on the IPSS score, subjects are grouped into Mild BPH (0–7), moderate BPH (8–19) and severe BPH (20–35).

The IPSS is commonly used in evaluation of BPH symptom severity. Also, since carcinoma of the prostate mimics BPH, all patients with LUTS are usually screened with serum total PSA. Those with elevated PSA or abnormal digital rectal examination findings or hypoechoic lesions on transrectal ultrasound undergo transrectal prostate biopsy. Other important parameters to be assessed as included in the CombAT study [13] include age, prostate volume, and maximum urine flow rate.

Cost effectiveness was calculated by dividing the difference in costs by the difference in health outcomes or quality- adjusted- life-year (QALY) saved between the two options of treatment to derive an incremental cost effectiveness ratio (ICER). A discount factor of 60 % [17] for both cost and effect was used.

We assumed that all patients included in this model have BPH which is a lifetime disease. All patients is assumed to enter the model in the early disease state of Mild BPH.

Every year, people with BPH either remain in a health state, move to a poorer health state or improve in their health state based on a transition probability as shown in Table 1. Table 2 shows baseline parameters depicting transition probabilities associated with BPH treatment in the model. The annual cost and utility values used in the model are shown in Tables 3 and 4 respectively. Table 5 is a summary of the cost outcomes and incremental cost effectiveness ratio for combination therapy compared with dutastaride monotherapy.

**Table 1:** A brief summary of cost details of service provision in the two tertiary institutions

	UNTH ($)			JUTH ($)		
	TURP	Drugs	Clinic	TURP	Drugs	Clinic
Surgical fees	153.67			165.76		
Anaesthetic drugs/items	64.71			117.64		
Antibiotic drugs	70.59			88.23		
Investigations	58.82			88.23		
Consumables	35.29			11.76		
Feeding fees	29.14			14.7		
Dutasteride (Avodart)		15.83/month			15.83/month	
Fixed dose tamsulosin- dutasteride (Duodart)		24/month			24/month	
Clinic consultations			47			47

**Table 2:** Baseline parameters showing transition probabilities associated with BPH treatment

Transitions (from-to)	Average annual probability	
Min, max	Dutasteride therapy	Combination therapy
Health to mild BPH	0.29	0.29
Mild BPH to moderate BPH	0.29	0.29
Moderate to severe BPH	0.16	0.058
Moderate/severe to AUR	0.13	0.04
Moderate/severe to TURP	0.16	0.056
TURP to repeat TURP	0.48	0.48
Severe to moderate BPH	0.46	0.49
Moderate to mild BPH	0.46	0.49
Death rate	0.013	0.013

**Table 3:** Baseline parameters showing annual costs and utility values associated with BPH treatment

Annual cost associated with treatment			
	Dutasteride US$	Fixed dose therapy US$	
Moderate BPH		338.82	Cost generated from cost data in two regional tertiary health centres in Nigeria
Severe BPH	223.53	338.82	
TURP	484.97	484.97	
AUR	29.41	29.41

**Table 4:** Baseline parameters showing utility values associated with BPH treatment

Utility and disutility values used in the Markov model		
	Utility values	Source
Mild	0.883	^a^Oppe et al. [26]
Moderate	0.787	
Severe	0.382	
TURP successful	0.833	
TURP repeat	0.833	
Disutility of AUR	−0.145	
Healthy	1.0	

Sensitivity test

^a^The quality of life was derived based on EQ-5D index score. Index score are based on general population variations survey that used TTO methods in Zimbabwe

**Table 5:** Cost, outcomes and incremental cost effectiveness ratio for combination therapy compared with dutasteride monotherapy

10-year horizon	Total cost US$	Total QALYs	Incremental costs	Incremental QALYs	Incremental cost per QALY gained
Fixed dose therapy	1450279504.76	18836849.43	594535937.61	401192.00	1481.92
Dutasteride	855743567.15	18435657.43			
15-year horizon					
Combination	2194123563.22	23890352.46	852086147.24	938291.33	908.13
* Dutasteride*	1578867548.20	20879011.27			

**Fig. 1:**
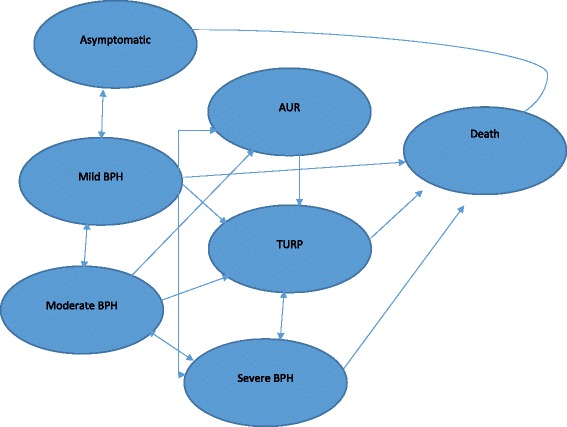
Illustrative Markov model showing discrete health states and direction of transitions (*arrows*)

The model structure used in this study is shown in Fig. 1. The model was programmed using Microsoft Excel (2007).

### Patient’s population

To calculate the cost effectiveness of fixed dose combination of dutasteride and tamsulosin versus dutasteride, a population of 2,862,363 who were 50 years of age based on the last published Nigeria’s population statistics were considered [18].

Healthy Subjects enter the model based on the published incidence rate of BPH in Nigeria. The 50 year age group was chosen because most patients become symptomatic at that age [4, 19].

### Outcome measures

The model estimates the following outcomes: cost of drug treatment, cost of consultation, Cost of AUR, cost of TURP, Cost of hospitalisation post TURP, and total quality adjusted life years (QALYs), incremental cost per life years gained, and incremental cost per QALY gained. Cost and outcomes were discounted at 6 % per annum based on the guidelines of World Health Organisation for developing countries.

### Treatment effects

The efficacy of the different interventions were derived from the CombaT clinical study report [13].

The Combination of Avodart and Tamsulosin (CombAT) study was a 4-years, multicentre, randomised, double-blind, parallel-group study in 4844 men ≥50 years of age with a clinical diagnosis of BPH, International Prostate Symptom Score ≥12, prostate volume ≥30 cm(3), prostate-specific antigen 1.5–10 ng/ml, and maximum urinary flow rate (Q(max)) >5 and ≤15 ml/s with minimum voided volume ≥125 ml.

### Quality of life

In the absence of data on utility values in Nigeria, we adopted the utility values derived from a Zimbabwe population perspective.

### Sensitivity test

This evaluation is subject to uncertainty relating to the data used in the study. The robustness of the ICER to variation can be ascertained by sensitivity analysis.

One-way deterministic sensitivity analysis (DSA) was performed to exclude deterministic, model structure and parameter uncertainties in the study.

The DSA was accomplished by changing the discount rates (0.01–0.06) while holding the remaining values in the model constant; to test the uncertainty around the model structure, treatment was commenced at health states, moderate BPH and severe BPH respectively and the variation from the baseline ICER noted. About 500, 000 people develop BPH annually in Nigeria. Though the Markov model was populated with about 570,000 patients at the age of 50 years, a deterministic sensitivity analysis was applied to both the duration of the cycle and number of patients fed to the model. The utility data used in the study were sourced from a country that has slightly different socioeconomic background, giving room to some degree of deterministic uncertainties. To address this, a thorough deterministic analysis was applied to the utility values. These values were varied by ±20 % keeping other parameters constant noting effect on baseline ICER. Also since cost data was primarily obtained from two tertiary institutions; which may introduce some deterministic uncertainties, these values were subjected to deterministic analysis by varying it by ±20 %.

Determining the Nigerian Government willingness to pay threshold was challenging because it was not available and could not be sourced from the literature. However, the 2012 Gross National Income per capita (GNI-PC) for Nigeria is US$2450 [20]. It has been suggested that twice per capita GNI-PC can be used as a reasonable threshold for determining cost effective analysis (8); therefore, the adopted threshold in this context was US$2450 per QALY saved for cost effective analysis of the new treatment.

### Ethical clearance

The model was programmed in Microsoft Excel. No ethics or consent were required for this study.

## Results

The discounted cumulative costs associated with FDCT and DM over 10 years were $US1.45 billion and US$855 million respectively; a difference of US$595 million.

The cumulative total of discounted QALYs associated with FDCT was 18.8 million over 10 years compared to 18.4 million for DM, a difference of 401192 QALYs. The calculated baseline ICERs were US$1481.92 per QALY gained.

### Sensitivity analysis

**Fig. 2:**
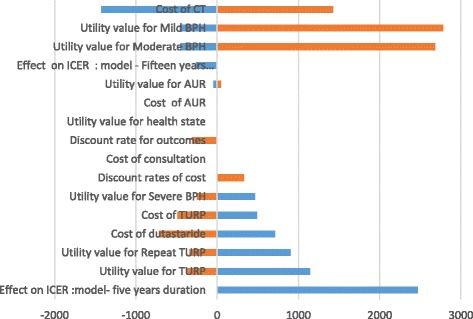
Graphical representation of the deterministic sensitivity analysis result; depicting effects of parameters on baseline ICER

The greatest impact on ICER (extremely sensitive) was seen when the assumption was altered and the model was run for 5 and 15 years duration. Also, significant impact was seen when the utility value for moderate BPH was altered by ±20 % (Fig. 2). Variation of the other parameters did not affect the ICER significantly. The effect of varying the health utility values by ±20 % is depicted in the graph. There appears to be little effect with the other utility parameters.

**Fig. 3:**
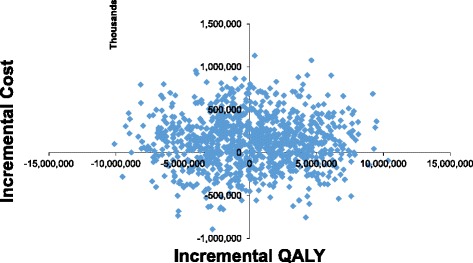
Cost effectiveness plane showing the plots of ICERs generated from simulation

Probabilistic analysis was carried out (1000 simulations) for both comparators. The cost effectiveness plane (Fig. 3) shows that most times FDCT was more costly but effective than DM as such most of the simulation plots are found in the North-East and North-west quadrant of the plane.

**Fig. 4:**
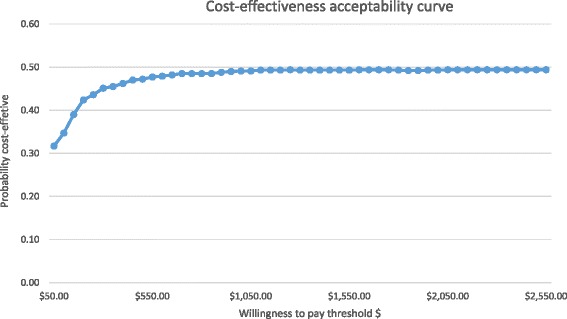
Cost- effectiveness acceptability curve for fixed dose combination therapy versus dutasteride

The CEAC [21] shows the probability that any intervention is cost effective conditional on the willingness to pay per QALY. The curves also illustrate the degree of uncertainty in the estimates. At a willingness to pay of US$2450 per QALY, the probability of FDCT being cost effective relative to DM was about 50 % (Fig. 4); which was same for the calculated baseline ICER value (US$1481.92 per QALY).

## Discussion

In this economic evaluation, cost effectiveness was calculated using the Markov modelling technique to aggregate information on cost and progression of disease in young adults. There was an assumption that the treatment effect of FDCT lasted for 10 years and this gave rise to an ICER value of US$1481.92 per QALY. However, extending the time horizon to 15 years, the ICER value reduced to US$ 908.13 per QALY. At 10 years’ time horizon the result obtained was similar to the results published by Ismaila et al. [22], though done in a different setting, which showed that FDCT was more costly and more effective than DM. Furthermore, as the time horizon was extended to 15 years the result obtained was comparable to other studies conducted in high income countries [22, 23]. The observed difference in ICER value between this study and similar studies in western countries could be explained by the low cost of BPH related surgeries in Nigeria.

A closer look at a similar study conducted in the setting of a Canadian population revealed a higher baseline ICER of CAN$49,414 per QALY [22] compared to this environment (US$1481.92 per QALY). The observed difference is a reflection of the difference in setting. Canada being a developed country with a higher GNI per capita than Nigeria. It is expected that this difference in GNI per capita may reflect cost of service provision in these two countries which could explain the difference in ICER. Also, in the context of the Nigeria’s health system, public health institution service delivery is subsidised by the government; though payment by the informal sector is primarily out of pocket. As such, service provision is still cheap compared to the more developed economies. This may explain why the baseline ICER is much lower than that of Canada.

In addition, in the 10 year horizon, about 281,761TURPs and 492,176 AURs were avoided in the FDCT group in our study. The total cost of treatment in the FDCT arm was higher than the DM group (difference of US$ 594.5 million). Despite this difference, FDCT was cost effective when the QALY -gained was put into consideration. A similar study on cost effectiveness of FDCT and tamsulosin monotherapy conducted by Geitona et al. [24] using the Greek health system perspective revealed that 1758 TURPs and 972 AURs were avoided by using FDCT over a 4 year horizon. Although, there was increase in disease management budget up to 7.9 % in 4 years, the study showed a reduction in costs associated with the overall treatment of the disease. In particular, savings associated with the use of combination therapy arose from the reduction in consultations, surgeries and AURs. These savings was estimated to be €1.95 million. These findings were comparable with ours, though the comparators were different (tamsulosin instead of DM); apparently a longer time horizon demonstrated clearly the effectiveness of FDCT [24].

On the other hand, with respect to QALY gained, about 2.8 million patients treated with FDCT in our study, gained 40,192 more QALYs than those treated with DM within the 10 year horizon. This is higher than the findings in the study by Bjerklund Johansen et al. [25] which showed that in a group of 100 patients treated for 4 years with FDCT, QALY gained was about 10 or 9 more QALYs than patients treated with dutasteride monotherapy. When the model time horizon was extended to the lifetime evaluation point, 100 patients treated with FDCT accrued about 16 QALYs more than those treated with DM. The observed difference is probably due to more number of patients being fed into our model compared to their study and the difference in model structure. However, that study [25] concluded that compared with the 4-year outcomes, the lifetime outcomes indicated that maintaining patients on combination therapy provided additional health benefits and a more favourable incremental cost-effective ratio than with dutasteride and was most likely to provide the greatest net monetary benefit at willingness to pay per QALY gained above £5400.

There appears to be no consensus for acceptable willingness to pay threshold in different regions. While the UK’s National Institute for Clinical Excellence has established a willingness-to-pay estimate that it applies to cost-effectiveness evaluations (roughly £20 000 per QALY gained), most European countries do not have clearly established willingness to pay threshold [25]. This observed challenge is also applicable to Africa. Although, there is no derived cost effectiveness threshold in Africa, our model predicts that ICERs for FDCT with dutasteride fall below thresholds that have been suggested in the literature, and thus FDCT with dutasteride is likely to be considered cost-effective for management. Unfortunately, currently, there are few works on cost effectiveness analysis on BPH treatment in Africa. Apparently, there appears to be no publication in the public domain on cost effectiveness of FDCT in BPH management in Africa based on Markov’s model. This may be due to the new introduction of FDCT. Some countries in Africa may not have adopted it as a treatment protocol. This study may form the basis of further studies in this area.

Furthermore, the sensitivity analysis showed that baseline ICER was significantly sensitive to duration of cycle. Taking a longer cycle (15 years) will result in reduction of ICER implying almost similar outcome in terms of cost for both FDCT and DM. Considering the life expectancy of Nigerian men which is about 55 years [20], adopting a life time horizon of 15 years, may not be realistic for our setting.

### Policy implication

Health insurance though introduced in Nigeria few years ago still grapples with the economic burden of providing full coverage. The major reason for this insufficient funding borders on depending mainly on contribution from the workers in formal sector (Social health insurance) and augmentation by the government. Effort to broaden the capital input is still in progress. Some health insurance packages are being explored currently to ensure enrolment of the informal sector.

Consequently, efficient use of the funds generated remains imperative. BPH is a chronic ailment that could span beyond a decade. Considering the vast population of Nigerians and the number of individuals who may eventually have BPH in their lifetime, any effort to reduce cost will interest policy makers.

Adopting FDCT though more expensive than DM is more effective. Based on the baseline ICER of US$1481.92, if policy makers should adopt a threshold ICER of US$2450 per QALY, it will be cost effective to adopt FDCT in the management of BPH in Nigeria.

With the current challenge of dwindling resources of mono-product economies such as Nigeria, cost-saving strategies and efficient utilisation of available funds cannot be over-emphasized. Adopting FDCT, with the afore-mentioned benefits, is a step in the right direction to ensure a sustainable and cost-effective management of BPH in this environment. This might encourage the SHI as it exists currently in Nigeria consider the inclusion of management of BPH in its benefit package.

Modelling to estimate treatment cost and effects; the model structure; quality of parameters used and the assumptions that have to be made are some of the limitations of this study.

First, it was assumed that most likely duration of drug effect was 10 years due to absence of long term data on effectiveness of FDCT. If the mean effect of FDCT was less than 10 years, the cost effectiveness of FDCT will have been overestimated.

Also, assuming homogenous health status for each health state; implying, transiting from a health state to another will be based on transition probabilities. However, the existence of co-morbidity in some of the patients that could make them transit faster to other state could not be controlled.

Inputting only direct cost can limit the utilisation of this study for serious policy decision making because, it lacks societal perspective. The use of utility values from a different country may not exactly represent the context of Nigeria. However, the effect of utility value on baseline ICER when subjected to DSA was very minimal. A detail CEA of FDCT is suggested in the future to include a robust model which captures the ideal utility values.

## Conclusion

Universal FDCT provision for Nigeria has major economic implications. This study in the context of its limitations has demonstrated the cost effectiveness of FDCT for the long term treatment of patients with moderate to severe BPH from the perspective of a developing country. Currently, there are few studies available to give economic data evidence to policy makers in Nigeria which is applicable to developing countries with similar economies. As such, the findings in this study will be relevant to policy makers in these countries.

## Abbreviations

BPH, benign prostatic hyperplasia; CEA, cost effective analysis; DM, dutasteride monotherapy; FDCT, fixed dose combination of dutasteride and tamsulosin; ICER, incremental cost effective ratio; QALY, quality adjusted life years
